# The impact of long-term oceanic warming on the Antarctic Oscillation in austral winter

**DOI:** 10.1038/s41598-017-12517-x

**Published:** 2017-09-26

**Authors:** Xin Hao, Shengping He, Huijun Wang, Tingting Han

**Affiliations:** 1 Nansen-Zhu International Research Center, Institute of Atmospheric Physics Chinese Academy of Sciences, Beijing, China; 2 Climate Change Research Center, Chinese Academy of Sciences, Beijing, China; 3 Collaborative Innovation Center on Forecast and Evaluation of Meteorological Disasters/Key Laboratory of Meteorological Disaster, Ministry of Education, Nanjing University of Information Science & Technology, Jiangsu, China; 4 University of Chinese Academy of Sciences, Beijing, China; 5 Geophysical Institute, University of Bergen and Bjerknes Centre for Climate Research, Bergen, Norway

## Abstract

Increasing greenhouse gas concentration and ozone depletion are generally considered two important factors that affect the variability of the Antarctic Oscillation (AAO). Here, we find that the first leading mode of sea surface temperature (SST) variability (rotated empirical orthogonal functions) shows a long-term upward trend from 1901 to 2004 and is closely related to the AAO index that is obtained using the observationally constrained reanalysis data. Further, regressions of the sea level pressure and the 500-hPa geopotential height anomalies, against the principle component associated with the long-term SST anomalies, display a seesaw behavior between the middle and high latitudes of the Southern Hemisphere in austral winter, which is similar to the high polarity of the AAO. The circulation responses to the long-term oceanic warming in three numerical models are consistent with the observed results. This finding suggests that the long-term oceanic warming is partly responsible for the upward trend of the AAO in austral winter. The thermal wind response to the oceanic warming in South Indian and South Atlantic Ocean may be a possible mechanism for this process.

## Introduction

The Antarctic Oscillation (AAO) is one of the most important extratropical atmospheric circulation modes in the Southern Hemisphere, and it has strong effects on the Southern ocean temperature, marine ecosystems and even climate variability in the Northern Hemisphere^1–5^. The AAO displays a seesaw behavior for the atmospheric mass between the middle and high latitudes of the Southern Hemisphere^6^. It can reflect the strength and position of the westerly winds over the mid-latitude that circle the Antarctic, following the mid-to-high-latitude atmospheric pressure gradient^7^.

The AAO has shown an increasing tendency in recent decades. Several studies suggested that anthropogenic greenhouse gas emissions and ozone play a critical role in the AAO trend^8–10^. This is due to the changes in direct radiative forcing by greenhouse-gas accumulation and stratospheric ozone depletion^8–10^. Cai *et al*.^8^ provided modeling evidence that the AAO exhibits a positive trend under increasing atmospheric CO_2_ concentration, but a reversed trend during the subsequent CO_2_ stabilization period. Shindell and Schmidt^9^ and Fyfe *et al*.^10^ indicated that both Antarctic ozone depletion and increasing greenhouses gases are responsible for the trend of the AAO in December–May. The response of the meridional temperature gradients in the stratosphere and upper troposphere to the ozone depletion and greenhouse gas abundances are generally considered the essential drivers for the change in the AAO trend, and the change in the AAO extends to the surface through the Antarctic vortex^8,9,11^.

Fogt and Bromwich^12^ and Ding *et al*.^13^ indicated that the AAO signature in the Pacific section resembles a Rossby wave train which is forced by the sea surface temperature (SST) in the tropical Pacific. Seviour *et al*.^14^ found that the Antarctic ozone depletion lead to a change in the AAO index, corresponding to a significant warming in the tropical oceans and a cooling in the middle latitudes of the Southern Hemisphere oceans. With the remarkable warming of global oceans, Staten *et al*.^15^ revealed that the oceanic warming induced by the variational radiative forcing may be the main driver of the zonal mean winds, inducing a significant increase in the AAO index. However, the key area of the oceanic warming and the associated mechanism are still unclear.

**Figure 1:**
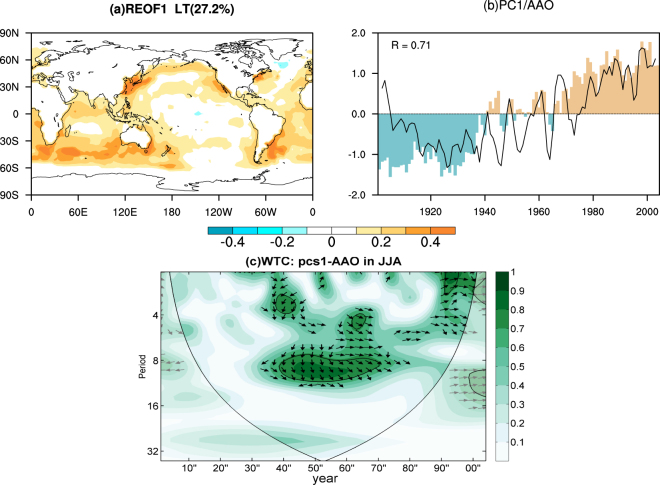
(**a**) Eigenvector of the first leading mode from the rotated EOF analysis of the annual mean SST, based on the period 1901–2004, for the global ocean. The value of explained variance is 27.2%. This mode reflects double the sea surface anomalies applied in the idealized experiments. (**b**) The PC1 associated with the long-term warming SST anomalies (bars) and normalized AAO index (line) for the period 1901–2004. (**c**) Wavelet coherence between the standardized AAO index and PC1 for period of 1901–2004. The 5% significance level against red noise is shown as a thick contour. The arrows show the phase relationship between the two series (with in-phase pointing right, out-phase pointing left). The map was generated by The NCAR Command Language (Version 6.3.0) [Software]. (2016). Boulder, Colorado: UCAR/UCAR/CISL/TDD. http://dx.doi.org/10.5065/D6WD3XH5.

Global oceans exhibited a long-term warming, especially in the South Indian Ocean and South Atlantic (Fig. 1a). This would generate a change in the surface temperature gradient in the southern hemisphere, which might contribute to a prominent change in AAO. Therefore, the objective of this study is to investigate the decisive influence of the long-term oceanic warming on the AAO in austral winter, based on reanalysis dataset and numerical experiments.

## Methods

We used a monthly mean meteorological reanalysis dataset gridded onto a 2° latitude–longitude grid, specifically the NOAA-CIRES 20th Century Reanalysis V2c since 1851, obtained from the NOAA National Climatic Data Center (https://www.esrl.noaa.gov/psd/data/gridded/tables/monthly.html). The AAO index is defined as the leading principal component of 850 hPa geopotential height anomalies south of 20°S^16^. The software related to wavelet coherence is provided by Aslak Grinsted (available online at http://noc.ac.uk/using-science/crosswavelet-wavelet-coherence). Additionally, numerical experiments performed by U.S. CLIVAR Drought Working Group are used, including both control experiments and idealized experiments with the NCAR Community Climate Model version 3 (CCM3) model, NCAR Community Atmosphere Model version 3.5 (CAM3.5) model, and Geophysical Fluid Dynamics Laboratory (GFDL) atmospheric model version 2.1 (AM2.1) model (available online at https://gmao.gsfc.nasa.gov/research/clivar_drought_wg/index.html).The CCM3 is a global spectral model with a horizontal T42 spectral resolution (approximately 2.8° latitude × 2.8° longitude), which is the atmospheric component of the NCAR Climate System Model^17^. The CCM3 includes a land surface model and an optional thermodynamic slab ocean and sea ice model. The CAM3.5 and GFDL AM2.1 are two state-of-the-art atmospheric general circulation models with a horizontal T85 spectral resolution (approximately 1.9° latitude × 2.5° longitude)^18^ and a horizontal resolution of 2° latitude × 2.5° longitude^19^, respectively. In the control runs, the models are forced by the monthly SST climatology (defined for the period of 1901–2004). The first leading pattern of SST variability, isolated from the annual mean SST of Hadley SST for the period of 1901–2004 by using rotated empirical orthogonal functions based on varimax rotation^20^, is multiplied by one standard deviation of the associated principal component (PC) to form a long-term oceanic warming anomalies (Fig. 1a). In the idealized experiments, the long-term SST anomalies (Fig. 1a) add to the monthly SST climatology to form a prescribed SST used in three models. In both the control runs and idealized runs, the SST forcing is repeated with no inter-annual variability for each year. The CAM3.5 and CCM3 experiments last for 51 years and the GFDL experiments last for 60 years. We identify circulation responses to the long-term SST anomalies by using composite and regression analyses.

## Results

### Driving role of long-term oceanic warming in the Antarctic Oscillation

To explore the potential impact of the long-term oceanic warming on the AAO in austral winter, we examine the relationship between the long-term SST anomalies and the spatio-temporal variability of the AAO. The PC associated with the long-term SST anomalies is closely related to the AAO index, with a correlation coefficient of 0.71 (Fig. 1b). It is not by chance that the AAO and long-term SST anomalies show an in-phase behavior since 1940 (Fig. 1c). As shown in Fig. 1b, the decrease in the AAO index corresponded to a long-term SST cooling during the period 1910–1925 and the increase in the AAO index was accompanied by a long-term SST warming after approximately 1930.

**Figure 2:**
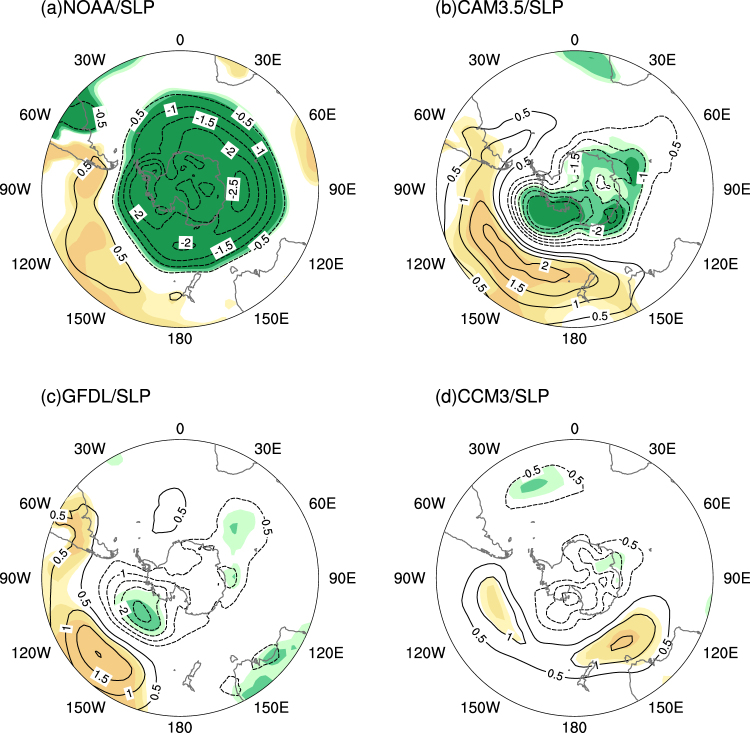
(**a**) Regression map of sea level pressure (contour, hPa) against PC1, for the period 1901–2004 in austral winter, based on the reanalysis dataset. The sea level pressure responses (contour, hPa) to the long-term SST anomaly pattern from (**b**) CAM3.5 model, (**c**) GFDL model and (**d**) CCM3 model in austral winter. The responses are the difference between the idealized run and control run. The light, medium, and dark shading indicates the 90%, 95% and 99% confidence levels (positive, yellow; negative, green), respectively. The map was generated by The NCAR Command Language (Version 6.3.0) [Software]. (2016). Boulder, Colorado: UCAR/UCAR/CISL/TDD. http://dx.doi.org/10.5065/D6WD3XH5.

Figure 2a depicts the regression maps of the PC against sea level pressure (SLP) derived from the NOAA dataset in austral winter. The positive values are found in the mid-latitudes of the Southern Hemisphere and significant negative values occur in the high latitude of the Southern Hemisphere, which is similar to the high polarity of the AAO. The responses in the three models are consistent with the spatial structure of the regression map, although the anomalies in the GFDL and CCM3 are weaker than in the CAM3.5. The SLP response to the long-term oceanic warming is characterized by an out-of-phase relationship between the middle and high latitudes, with large positive anomalies over the south Pacific, and negative anomalies over the whole Antarctic Peninsula. It suggests that the oceanic warming induces a positive AAO anomaly (Fig. 2b–c).

**Figure 3:**
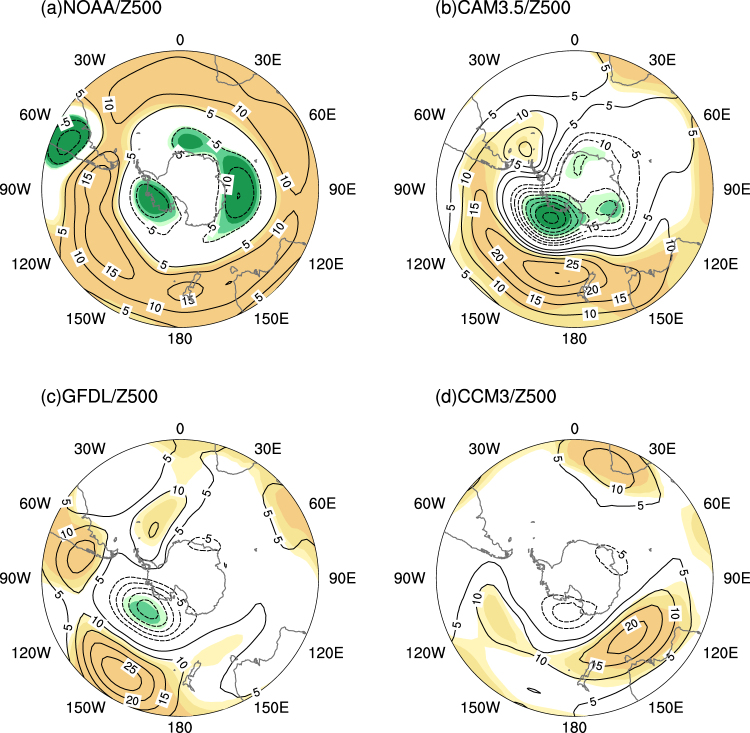
(**a**) Regression map of 500-hPa geopotential height (contour, m) against PC1, for the period 1901–2004, in austral winter based on the reanalysis dataset. 500-hPa geopotential height responses (contour, m) to the long-term SST anomaly pattern from (**b**) CAM3.5 model, (**c**) GFDL model and (**d**) CCM3 model in austral winter. The responses are the difference between the idealized run and control run. The light, medium, and dark shading indicates the 90%, 95% and 99% confidence levels (positive, yellow; negative, green), respectively. The map was generated by The NCAR Command Language (Version 6.3.0) [Software]. (2016). Boulder, Colorado: UCAR/UCAR/CISL/TDD. http://dx.doi.org/10.5065/D6WD3XH5.

Based on the reanalysis dataset, the regression of the 500-hPa geopotential height against the PC displays that the positive coefficients in the middle latitudes surround the negative coefficients in the high latitudes (Fig. 3a). The strongest positive center is found in the middle latitude of the Pacific Ocean, and two negative centers are in Siple Island and the polar latitude of the Indian Ocean. The spatial distribution of the 500-hPa geopotential height responses in the three models is similar to the results obtained from the observationally constrained reanalysis dataset (Fig. 3). The CAM3.5 and GFDL effectively capture the largest positive center over the middle latitude of the Pacific Ocean and one polar negative center in Siple Island (Fig. 3b and c). However, the negative anomalies over the high latitude of the Southern Hemisphere, as detected in the CCM3 model, are not statistically significant (Fig. 3d). These evidences reveal that the long-term oceanic warming may be one of major contributing factors to the variability of the AAO in austral winter. This finding provides new knowledge on the interaction between the oceans and the AAO, which is important for understanding the oceanic forcing on climate change.

## Discussion

**Figure 4:**
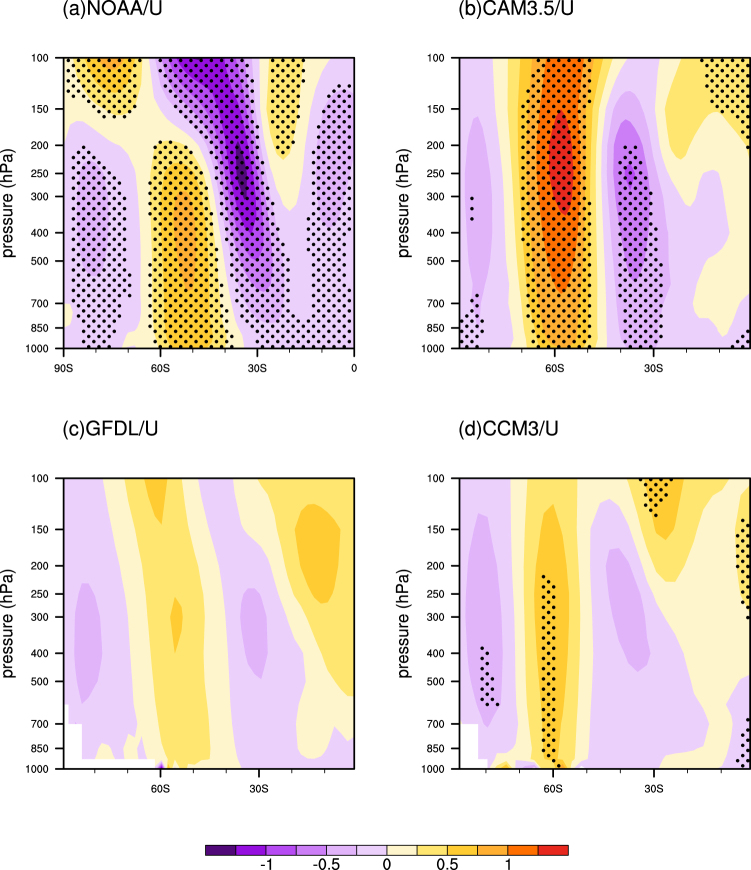
(**a**) Regression map of zonally averaged zonal wind (contour, m/s) against PC1, for the period 1901–2004, in austral winter, based on the reanalysis dataset. The zonally averaged zonal wind responses (contour, m/s) to the long-term SST anomaly pattern from (**b**) CAM3.5 model, (**c**) GFDL model and (**d**) CCM3 model in austral winter. The responses are the difference between the idealized run and control run. The dotted areas indicate the 90% confidence levels.

The high index polarity of the AAO is accompanied by stronger-than-average westerlies in 50°S–70°S and weaker-than-average westerlies in 30°S–50°S^4,7^. And the zonal wind anomalies tend to be strongest at 35 and 55 degrees latitude, with a node of ~45 degrees^7^. The warming trend of the long-term SST anomalies mostly emerges in the Indian Ocean and Atlantic Ocean and the warmest anomalies located around 45°S (Fig. 1a). The abnormal distribution of thermal force leads to a decrease in the equator-to-midlatitude temperature gradient and an increase in the midlatitude-to-pole temperature gradient and, consistent with thermal wind balance, easterly anomalies in 30°S–45°S and westerly anomalies in 50°S–65°S (Fig. 4a and S2). It tends to be a high index polarity of the AAO. The results of the three models are in good agreement with the NOAA results, although the positions of nodes are slightly different (Fig. 4b–c). The zonal wind perturbations forced by the long-term SST anomalies extend upward from the surface into the tropopause, which can enhance upward convergence movement at the subpolar low-pressure belt and subsidence divergence at the subtropical high-pressure belt. Therefore, the long-term SST anomalies favor positive SLP anomalies in the middle latitudes and negative SLP anomalies in the high latitudes (Fig. 2). These evidences indicate that a north-south (“− +”) seesaw in the zonal wind anomalies is driven by the long-term SST anomalies, thereby increasing the AAO. Additionally, we note that the convective heating in the three models are different, which directly affect the magnitude of the thermal wind. Thus, we consider the different convective schemes in the three models may be responsible for the differences of the circulation responses in models.

Recent studies have shown that increasing greenhouse gas accumulation, stratospheric ozone depletion and long-term warming in tropical Pacific SST lead to an upward trend of the AAO^8–10,13^. As noted in Shindell and Schmidt^9^ and Fyfe *et al*.^10^, the AAO anomaly induced by greenhouse gas accumulation or stratospheric ozone depletion is zonally symmetric. The spatio-temporal variability of the long-term oceanic warming, which is consistent with previous studies, is attributed to the increasing atmospheric greenhouse gases due to the change in radiative forcing^21–24^. However, the long-term oceanic warming provides a zonally asymmetric forcing for the AAO. For the foregoing reasons, the results from the NOAA reanalysis may reflect the superposition of all relevant factors forced variability, while the responses of the AAO in the three models only reflect the long-term SST anomalies forced variability. This may be the reason why the response is so zonally asymmetric in the models compared to the NOAA reanalysis.

## Electronic supplementary material

Supplementary information
