# Supplementary material for: The impact of long-term oceanic warming on the Antarctic Oscillation in austral winter
Source: Sci Rep. 2017 Sep 26;7:12321. doi: 10.1038/s41598-017-12517-x (PMC5615052; doi:10.1038/s41598-017-12517-x)
Supplement: Supplementary file 1 — Supplementary information [file 41598_2017_12517_MOESM1_ESM.pdf]

Supplementary Material

Article Title: The impact of long-term oceanic warming on the Antarctic Oscillation in austral winter

Authors: Xin Hao, Shengping He, Huijun Wang and Tingting Han

Contents:

1) Leading role of warming oceans in the air-sea interaction in the middle latitude of the Southern Hemisphere (Long-term SST dataset obtained from the Extended Reconstructed SST v4 since 1985: https://www.esrl.noaa.gov/psd/data/gridded/data.noaa.ersst.v4.html)

2) Figure S1: Correlation coefficient between the upward heat flux and local SST variability for the period of 1901—2004 in austral winter.

1) Leading role of warming oceans in the air-sea interaction in the middle latitude of the Southern Hemisphere

Correlation between upward heat flux anomalies (the sum of upward sensible heat flux anomalies and upward latent heat flux anomalies) and local SST anomalies reflects the leading forcing in air-sea interaction (Cayan 1992). Positive correlation between upward heat flux anomalies and local SST anomalies suggests a driving role of SST on the atmospheric circulation. Fig. S1 shows the relation of upward heat flux to the local SST anomalies, and positive values are agreement well with the largest warming SST in 30°S-60°S. It implies that long-term warming SST anomalies can drive changes in atmospheric circulation in mid-latitudes of the Southern Hemisphere.

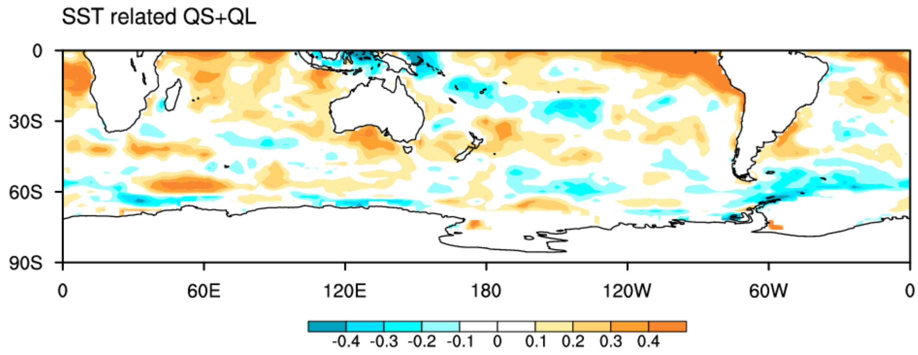

Fig. S1 Correlation coefficient between the upward heat flux and local SST variability for the period of 1901—2004 in austral winter. The map was generated by The NCAR Command Language (Version 6.3.0) [Software]. (2016). Boulder, Colorado: UCAR/UCAR/CISL/TDD. http://dx.doi.org/10.5065/D6WD3XH5.

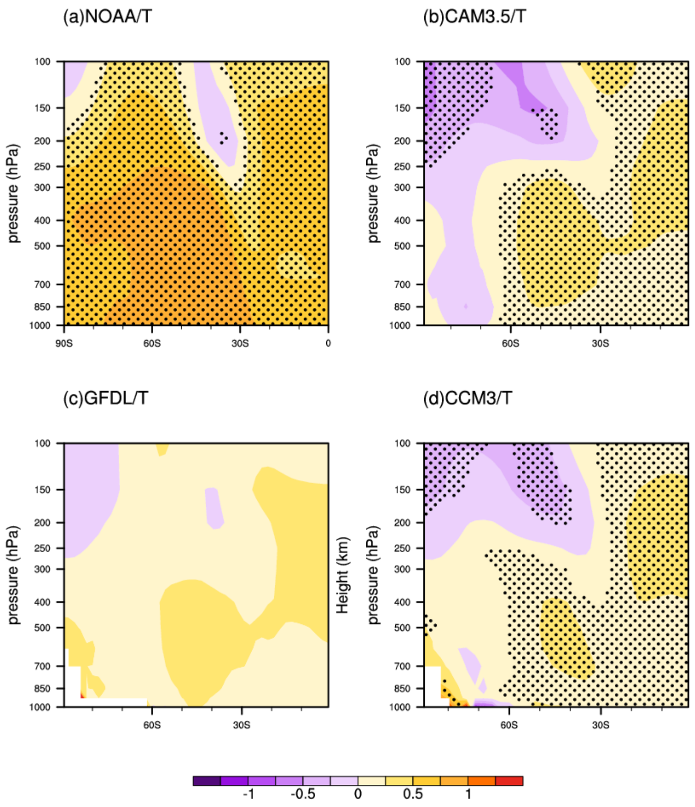

Fig. S2 (a) Regression map of zonally averaged air temperature (contour) against PC1, for the period 1901–2004, in the austral winter, based on the reanalysis dataset. The zonally averaged air temperature responses (contour) to the long-term SST anomaly pattern from (b) CAM3.5 model, (c) GFDL model and (d) CCM3

model in austral winter. The responses are the difference between the idealized run and control run. The dotted areas indicate the 90% confidence levels.

References:

Cayan, 1992, Latent and Sensible Heat Flux Anomalies over the Northern Oceans: Driving the Sea Surface Temperature.
